# Sestrin2 in cancer: a foe or a friend?

**DOI:** 10.1186/s40364-022-00380-6

**Published:** 2022-05-08

**Authors:** Moein Ala

**Affiliations:** School of Medicine, Tehran University of Medical Sciences (TUMS), Tehran, Iran

**Keywords:** Sestrin2, Cancer, Oxidative stress, Autophagy, Apoptosis, mTORC1

## Abstract

Sestrin2 is a conserved antioxidant, metabolism regulator, and downstream of P53. Sestrin2 can suppress oxidative stress and inflammation, thereby preventing the development and progression of cancer. However, Sestrin2 attenuates severe oxidative stress by activating nuclear factor erythroid 2-related factor 2 (Nrf2), thereby enhancing cancer cells survival and chemoresistance. Sestrin2 inhibits endoplasmic reticulum stress and activates autophagy and apoptosis in cancer cells. Attenuation of endoplasmic reticulum stress and augmentation of autophagy hinders cancer development but can either expedite or impede cancer progression under specific conditions. Furthermore, Sestrin2 can vigorously inhibit oncogenic signaling pathways through downregulation of mammalian target of rapamycin complex 1 (mTORC1) and hypoxia-inducible factor 1-alpha (HIF-1α). Conversely, Sestrin2 decreases the cytotoxic activity of T cells and natural killer cells which helps tumor cells immune evasion. Sestrin2 can enhance tumor cells viability in stress conditions such as glucose or glutamine deficiency. Cancer cells can also upregulate Sestrin2 during chemotherapy or radiotherapy to attenuate severe oxidative stress and ER stress, augment autophagy and resist the treatment. Recent studies unveiled that Sestrin2 is involved in the development and progression of several types of human cancer. The effect of Sestrin2 may differ depending on the type of tumor, for instance, several studies revealed that Sestrin2 protects against colorectal cancer, whereas results are controversial regarding lung cancer. Furthermore, Sestrin2 expression correlates with metastasis and survival in several types of human cancer such as colorectal cancer, lung cancer, and hepatocellular carcinoma. Targeted therapy for Sestrin2 or regulation of its expression by new techniques such as non-coding RNAs delivery and vector systems may improve cancer chemotherapy and overcome chemoresistance, metastasis and immune evasion that should be investigated by future trials.

## Introduction

Sestrin2 is a conserved antioxidant and metabolic regulator which can markedly reprogram intracellular signaling pathways [1]. Recently, numerous studies revealed that Sestrin2 vigorously alleviates cellular damage and mitigates organ dysfunction in response to harmful and noxious stimuli [2–4]. Primarily, Sestrin2 has been introduced as a downstream of P53, a major oncosuppressor protein [5, 6]. It was assumed that the tumor-suppressing effect of P53 is partly mediated via Sestrin2 [7]. For instance, it was shown that P53 increases Sestrin2 expression to confine UVB damage to keratinocytes [8]. Further, it has been proposed that mutant P53 accelerates oxidative stress in cancer cells, thereby modulating several signaling pathways [9]. Consistently, P53 mutation can significantly downregulate Sestrin2 expression in tumor cells [10]. However, Sestrin2 can also be induced independently of P53 [11].

Several cellular stress conditions such as increased levels of oxidative stress, ER stress, and hypoxia can stimulate Sestrin2 expression which can compensate for the damage and provide cytoprotection [12]. Similarly, the Sestrin2 level is usually elevated in damaged organs, compared to a healthy condition. Also, its expression correlates with the severity of the damage [13]. Moreover, Sestrin2 can regulate the function of mitochondrial complexes and improve mitochondrial function in response to hypoxia and cellular stress [14]. It modulates cellular metabolism partly through enhancing mitochondrial biogenesis [15].

In recent years, it was shown that Sestrin2 can regulate cell proliferation and cell death [16, 17]. Several studies uncovered that Sestrin2 is heavily involved in the pathophysiology of cancer and can significantly contribute to predicting the prognosis and clinical course of cancers [18–20]. Herein, this review discusses the involvement of Sestrin2 in cancers with an emphasis on the underlying molecular mechanisms.

## Oxidative stress and cancer

Chronic exposure to inflammatory response is a major driver of cancer development. Inflammation and oxidative stress can vigorously damage DNA structure and contribute to tumorigenesis and malignant transformation [21]. Also, impaired antioxidant defense contributes to carcinogenicity [22]. Oxidative stress not only helps the development of cancer but a minimum level of oxidative stress was shown to be pivotal for viability, migration, and aggressive behavior of cancer cells [21, 23]. An adequate amount of ROS is essential for tumor cell cycle progression and proliferation [24]. Although, several chemotherapeutic agents can severely activate oxidative stress which results in cancer cells apoptosis [25–27]. Intriguingly, tumor cells can develop mechanisms to attenuate oxidative stress induced by chemotherapeutic agents. These mechanisms are involved in tumor cells chemoresistance and pharmacological intervention to attenuate them improves chemoresistance [26–28]. Oxidative stress is a contributory factor for both the initiation and progression of cancer but excessive activation of oxidative stress known as the burst of oxidative stress shortens cancer cells viability [21, 22, 25]. Therefore, oxidative stress is a double-edged sword in relation to cancer cells and can be meticulously manipulated to favorably improve cancer chemotherapy.

## Sestrin2 interacts with tumor suppressors and oncoproteins

Sestrin2 interacts with numerous components of intracellular signaling pathways and can differently alter cancer cells behavior by modulating each one of these pathways.

### Sestrin2, Nrf2 and cancer

**Fig. 1:**
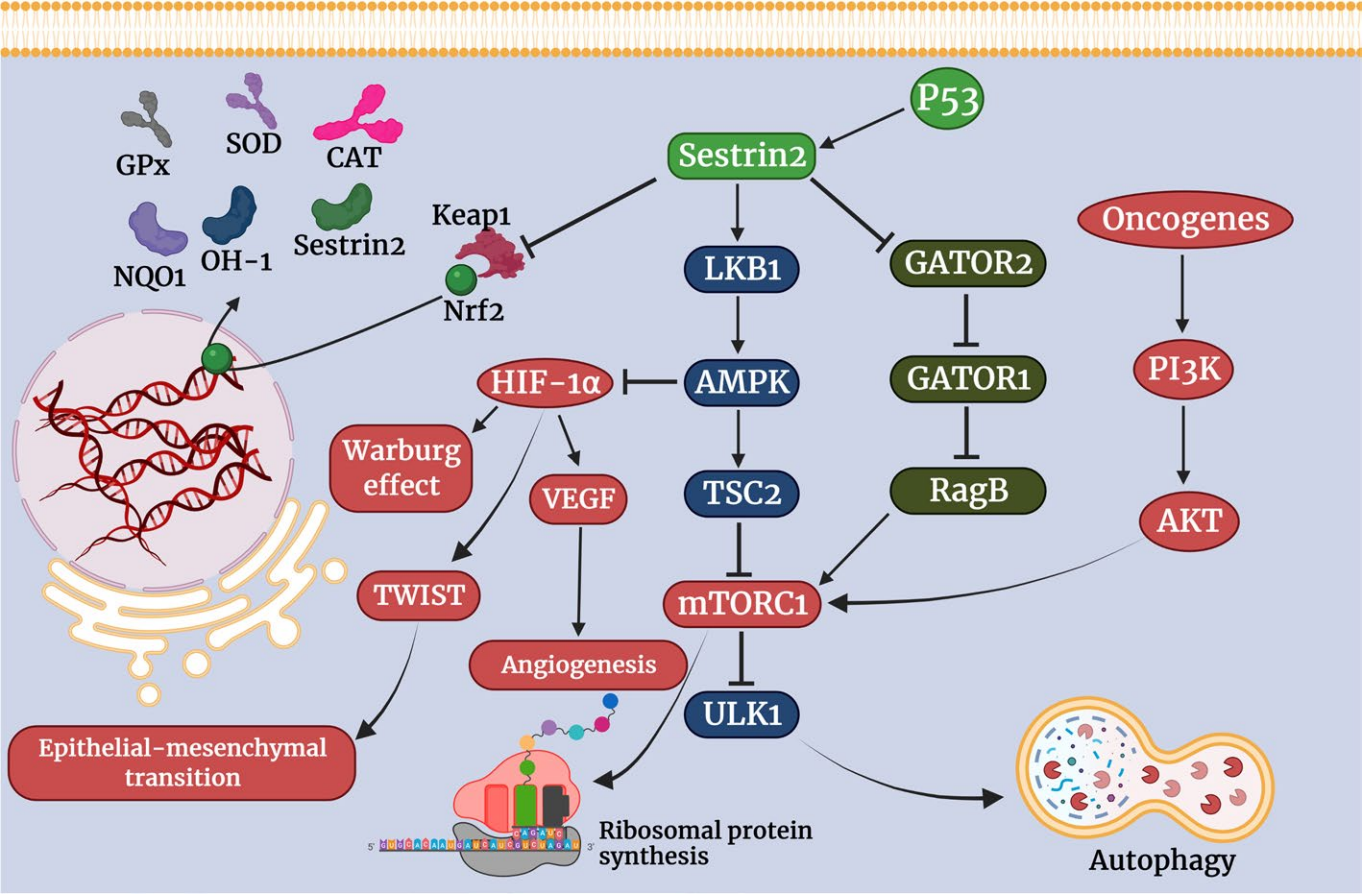
The interactions between Sestrin2 and different signaling pathways in cancer. P53 is a major upstream molecule for Sestrin2. Oxidative stress and P53 can increase Sestrin2 expression. In response, Sestrin2 liberates Nrf2 from Keap1 sequestration. Activated Nrf2 binds to ARE and promotes the gene expression of several antioxidants such as Sestrin2, Ho-1, NQO1, GSH, SOD, GPx, and CAT. Sestrin2 is a leucine sensitive factor for mTORC1. Sestrin2 attenuates the inhibitory effect of GATOR2 on GATOR1. GATOR1 suppresses RagB to inhibit mTORC1. Further, Sestrin2 activates the LKB1/AMPK pathway which can inhibit mTORC1 through TSC2. mTORC1 inhibition can enormously abrogate the oncogenic effects of the PI3K/AKT/mTORC1 pathway. mTORC1 inhibition facilitates autophagy and impedes ribosomal protein synthesis. Several of these proteins are needed for cancer cells metabolism and cell cycle progression and their absence halts cancer cells proliferation. Activation of the LKB1/AMPK pathway can also effectively downregulate HIF-1α which is involved in intra-tumor angiogenesis, Warburg effect, epithelial-mesenchymal transition, and tumor cells immune evasion

Nrf2 is a transcription factor for several antioxidants that binds to antioxidant response element (ARE) within SESN2 and genes of other antioxidants such as glutathione, heme oxygenase (HO-1), nicotinamide adenine dinucleotide phosphate (NADP)-H dehydrogenase 1 (NQO1), *superoxide dismutase (SOD),* catalase (CAT), glutathione peroxidase (GPx), thioredoxin reductase (TrxR) and peroxiredoxins [29–31]. Sestrin2 can liberate Nrf2 from Keap1 anchoring, prevent its degradation and activate it (Fig. 1) [31, 32]. Nrf2 plays dual roles in cancers. It was shown that Nrf2 particularly its short-term activity prevents cancer development in mice exposed to carcinogenic stimuli [33, 34]. In contrast, Nrf2 hyperactivation protects cancer cells against oxidative stress, increases their proliferation and invasive behavior, and enhances their resistance to chemotherapy and radiotherapy [35, 36]. Nrf2 can also reprogram cancer cells metabolism. It facilitates fatty acids β-oxidation and pentose phosphate pathway [37]. Nrf2 enhances nucleotide synthesis and amino acids bioavailability [37]. These alterations improve cancer cells energy metabolism and provide more substrates for the synthesis of organic macromolecules and proliferation [37]. However, transient activation of Nrf2 attenuates oxidative stress and prevents carcinogenesis, sustained activation or overactivation of Nrf2 contributes to cancer cells viability and proliferation [38]. In other words, Nrf2 prevents cancer development but may assist the currently developed cancer.

### Sestrin2, mTORC1 and cancer

Phosphoinositide 3-kinases (PI3K)/protein kinase B (AKT)/mammalian target of rapamycin complex 1 (mTORC1) is one of the most important oncogenic pathways in different cancers [39]. mTORC1 activation is crucial for ribosomal protein synthesis and cancer cells growth. Without a sufficient amount of protein synthesis cancer cells cannot effectively respond to their metabolic and proliferative needs [40, 41]. Furthermore, mTORC1 is a negative regulator of autophagy [42]. mTORC1 inhibits unc-51 like autophagy activating kinase (ULK) and other activators of autophagy [43]. mTORC1 inhibition leads to cancer cell cycle arrest, activates autophagy and apoptosis, and reduces cancer cells viability [43, 44]. mTORC1 inhibition can also effectively abrogate cancer cells chemoresistance and enhance the effect of other chemotherapeutic agents [40]. Furthermore, inhibitors of PI3K/AKT/mTORC1 particularly mTORC1 inhibitors such as sirolimus and everolimus are currently used for preclinical studies and clinical trials of different cancers [39].

Interestingly, it was shown that P53 acts through Sestrin2 to inhibit mTORC1 and exert parts of its tumor-suppressing effects [42]. Sestrin2 enhances liver kinase B1 (LKB1)-mediated activation of AMP-activated protein kinase (AMPK), thereby regulating cellular metabolism [45]. Insufficient or low nutrient condition activates LKB1/AMPK, resulting in growth arrest. On the contrary, hyperglycemia and overnutrition downregulate LKB1/AMPK and is associated with a higher risk of cancers in obese and diabetic patients [46]. Overactivation of AMPK may prevent or restrict cancer cells proliferation but sufficient function of AMPK is also needed for cancer cells viability (Fig. 1) [47].

**Fig. 2:**
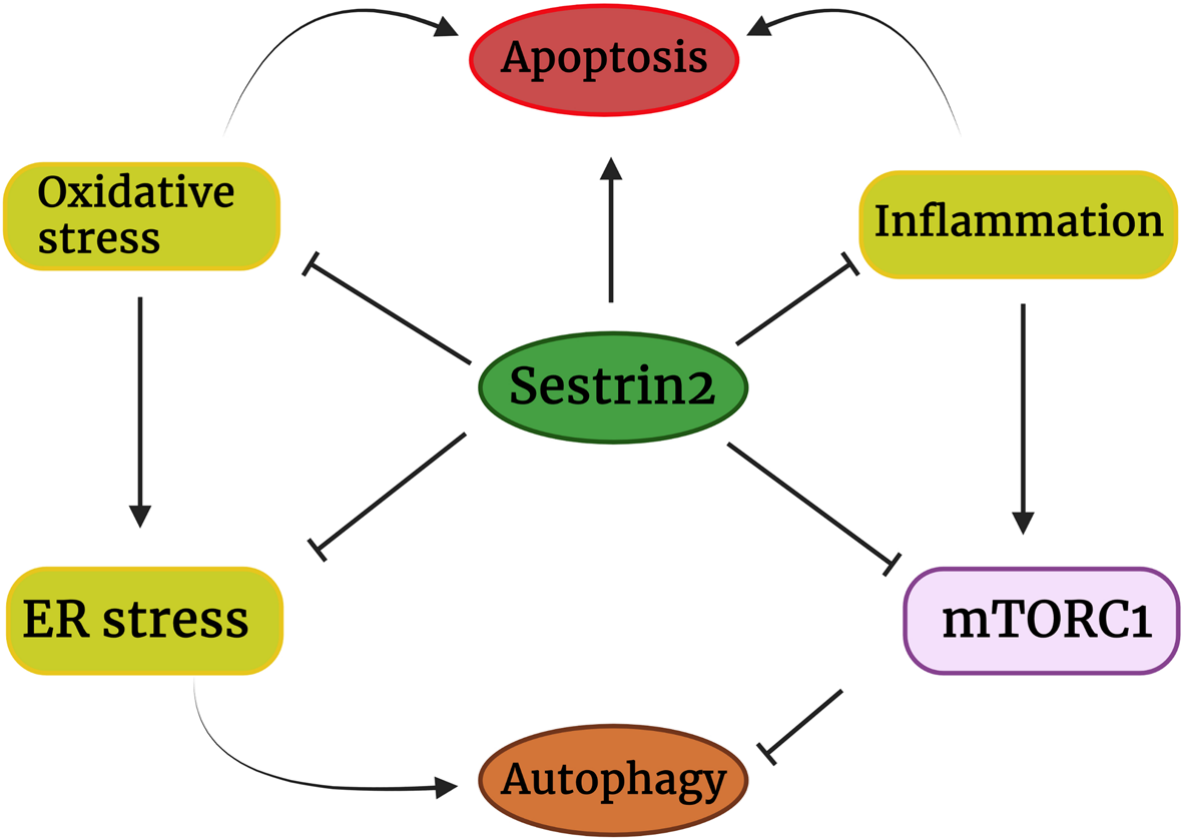
The involvement of Sestrin2 in oxidative stress, inflammation, ER stress, autophagy, and apoptosis of cancer cells. Sestrin2 can attenuate oxidative stress and inflammation and directly or indirectly inhibit ER stress. In return, inflammation, oxidative stress and ER stress upregulate Sestrin2 to ameliorate the damage. Severe activation of ER stress and oxidative stress leads to excessive activation of autophagy and apoptosis. This can shorten cancer cells survival. However, mild to moderate activation of ER stress may enhance cancer cells survival by activating autophagy. Autophagy allows cancer cells to recover in stress conditions, while excessive activation of autophagy leads to cancer cells death. Although Sestrin2 inhibits ER stress, it can promote autophagy via downregulation of mTORC1. Furthermore, it has been observed that Sestrin2 can induce apoptosis in cancer cells, independent of its effects on autophagy, ER stress, and oxidative stress

Sestrin2 activates AMPK and thereby tuberous sclerosis complex (TSC1) 1/TSC2 to inhibit mTORC1 [42]. Additionally, recent studies uncovered that Sestrin2 can attenuate mTORC1 through GATORs. Herein, Sestrin2 inhibits GATOR2 and attenuates its inhibitory effect on GATOR1. GATOR1 inactivates RagB, a GTPase necessary for mTORC1 activation. Hence, Sestrin2 can release GATOR1 and enhance its inhibitory effect on mTORC1 [48]. It was shown that Sestrin2 is a leucine-sensitive regulator of GATOR2 and mTORC1 [49]. In the presence of leucine deficiency, ULK1 phosphorylates Sestrin2 and strengthens its interaction with GATOR2 to liberate GATOR1 and inhibit mTORC1. In contrast, leucine sufficiency dephosphorylates Sestrin2 and leads to mTORC1 activation [49]. It may partly justify the inhibitory effect of nutrient deficiency and hunger signal on mTORC1 [50]. Consistently, it was shown that Sestrin2 can strongly inhibit mTROC1 and is a positive regulator of autophagy [51]. mTORC1 inhibition has a great impact on cancer cells and is responsible for a major proportion of Sestrin2 effects on cancers (Figs. 1 and 2).

### Sestrin2, HIF-1α and cancer

HIF-1α is a transcription factor and an oncoprotein that can increase tumor cells mobility and metastasis [52]. Consistently, it was unveiled that specific polymorphisms of HIF-1α are associated with cancer susceptibility and progression [53]. Further, HIF-1α overexpression prognosticates the poor prognosis of cancer [54]. HIF-1α increases vascular endothelial growth factor (VEGF) to enhance intra-tumor angiogenesis [52]. It also induces TWIST gene expression and accelerates epithelial-mesenchymal transition (EMT) [52]. Additionally, HIF-1α enhances the gene expression of several enzymes to improve glucose consumption and energy metabolism in tumor cells, known as aerobic glycolysis or the Warburg effect [55]. Moreover, HIF-1α is involved in tumor cells immune evasion [56]. Sestrin2 can facilitate HIF-1α degradation via AMPK which can contribute to confine cancer cells invasion and metastasis [57]. AMPK can negatively regulate aerobic glycolysis by downregulating HIF-1α [58]. Sestrin2 can impair several dimensions of tumor cells biology by downregulating HIF-1α including angiogenesis, metastasis, and immune evasion (Fig. 1).

## Sestrin2, inflammation, and cancer

The interplay between inflammation and carcinogenesis has been elucidated by many studies [59]. Marjolin ulcer and higher prevalence of colon cancer in patients with inflammatory bowel disease are proofs-of-concept in this regard [60, 61]. Inflammation activates different signaling pathways which are simultaneously involved in the neoplastic transformation of normal tissue and provides the prerequisites for cancer development [59, 62]. Because of the high similarity between carcinogenic pathways and regenerative pathways, increased tissue regeneration during inflammation increases the risk of cancer development [63]. Furthermore, cancer cells depend on the inflammatory response for proliferation, angiogenesis, immune evasion, invasion, and chemoresistance [62, 64]. Targeting the mutual interaction between inflammation and cancer can break the futile cycle and increase the efficacy of chemotherapy [62].

Sestrin2 is a major negative regulator of inflammation and SESN^−/−^ mice are susceptible to more severe inflammatory responses and show impaired wound healing [65]. Sestrin2 can suppress M1 macrophage-related inflammatory response [66]. It attenuates toll-like receptors (TLR)-mediated inflammatory response, inhibits the c-Jun N-terminal kinases (JNK) signaling pathway in inflammation, and prevents the release of several inflammatory cytokines such as interleukin (IL)1β, IL6, IL17A, interferon γ (INF-γ), and tumor necrosis factor α (TNF-α) [66, 67]. JNK activation is involved in the cell cycle progression, chemoresistance, and survival of cancer cells [68]. Inflammatory cytokines play crucial roles in cancer biology. For instance, it was shown that IL1 is usually overexpressed in metastases of several types of human cancer and positively correlates with VEGF expression. Also, an IL1 receptor antagonist markedly decreased xenograft growth in IL1-producing tumors [69]. Similarly, it was observed that IL17A polymorphisms can strongly affect the risk of gastric cancer [70]. Cancer cells produce IL17A which helps their proliferation and chemoresistance [71].

Furthermore, Sestrin2 can inhibit nuclear factor kappa B (NF-κB) which is a major driver for inflammatory response and promotes the gene expression of numerous inflammatory mediators [67]. Previously, it has been reported that NF-κB signaling can contribute to cancer development and angiogenesis and increase cancer cells motility [64, 72]. Besides, Sestrin2 can inhibit the NLR family, pyrin domain containing 3 (NLRP3) inflammasome [73]. NLRP3 inflammasome activation can increase the proliferative and metastatic capacity of cancer cells [74, 75]. Here, it was mentioned how Sestrin2 can suppress several inflammatory signaling pathways. The anti-inflammatory properties of Sestrin2 can impair signaling pathways needed for tumorigenesis and tumor progression.

## Sestrin2, autophagy, and cancer

Previously, it was noted that Sestrin2 can activate autophagy, particularly through inhibition of mTORC1 [51]. Similar to oxidative stress, autophagy is assumed to play dual roles in cancer [76]. Autophagy is in close relation with apoptosis and is sometimes remembered as type II programmed cell death [77]. Currently, it is assumed that excessive and uncontrolled activation of autophagy can damage cancer cells and help cancer treatment [78]. Similarly, rapamycin and curcumin can decrease cancer growth by inhibiting mTORC1 which is associated with autophagy activation [79].

In contrast, pharmacological blockade of autophagy by chloroquine and other inhibitors of autophagy was shown to decrease cancer cells growth and increase their apoptosis [80–82]. To clarify this ambiguity, Ishibashi et al. and Gong et al. showed that chloroquine can increase the cytotoxic activity of rapamycin on osteosarcoma cells and acute lymphoblastic leukemia cells by inhibiting autophagy and increasing apoptosis [83, 84]. A similar scenario was observed for other anti-cancer agents with the capability of inducing autophagy [81, 82]. Attenuation of autophagy increases apoptosis and the anti-cancer property of mTORC1 inhibitors [83]. Also, trehalose, a non-mTORC1 inhibitor autophagy activator despite rapamycin, as a mTORC1 inhibitor autophagy activator, increased prostate cancer cells survival against docetaxel-induced apoptosis [85]. These findings show that the beneficial effects of mTORC1 inhibitors against cancer cells may be mediated by attenuating other functions of mTORC1 that are not related to autophagy. Potentiation of autophagy may be an unwanted effect of mTORC1 inhibition and simultaneous inhibition of autophagy can strongly enhance the anti-tumor efficacy of mTORC1 inhibitors.

Inhibition of autophagy can impair the restorative capacity of cancer cells and subsequent accumulation of damaged organelles and misfolded proteins accelerates apoptosis [81]. Autophagy also augments cancer cells stemness and postpones senescence [86]. Tumor cells can use autophagy as a defense mechanism against chemotherapy agents, thereby developing chemoresistance [87, 88]. However, there are studies that have shown that silencing or downregulation of autophagy-related proteins such as autophagy-related 5 (ATG5) can impair autophagy and prevent cancer cells death [89, 90]. Also, it was shown that Sestrin2 can increase cancer cells death in hepatocellular carcinoma, osteosarcoma, colorectal cancer, and bladder cancer cells via autophagy flux [7, 91–94].

Autophagy has a preventive effect on the development of cancer. Autophagy can resolve the damages exerted by oxidative stress and inflammation and protect DNA or other cell components against oncogenic alterations [95, 96]. It was shown that inhibition of autophagy increases the number of oncogenic foci while slowing down cancer progression after development in the mice model of lung cancer [95]. Therefore, autophagy can initially prevent the incidence of cancer although, it may help or prevent cancer progression in later stages. Sestrin2, as an inducer of autophagy, can help to decrease the development of cancers but may enhance or impede cancer progression depending on the condition. Several studies revealed that Sestrin2-mediated autophagy flux is associated with decreased tumor growth, however, Sestrin2 has several functions, and augmentation of autophagy is just one of them.

## Sestrin2, ER stress and cancer

ER stress upregulates Sestrin2 through unfolded protein response activators such as protein kinase RNA-like endoplasmic reticulum kinase (PERK) and inositol-requiring enzyme 1 (IRE1) and activating transcription factor 6 (ATF6) [97, 98]. In return, Sestrin2 attenuates ER stress to alleviate inflammation [97]. Also, SESN2 knockdown was shown to increase unfolded protein response and ER stress, in response to oxidative stress or inflammatory response [99, 100]. ER stress can also increase inflammatory response and accelerate tumorigenesis [101]. ER stress links oxidative stress and inflammation to autophagy which can influence cancer cells viability [101]. In particular, hypoxia upregulates ER stress and thereby autophagy to increase tumor cells survival in the stressful microenvironment [102].

In contrast, excessive ER stress and autophagy of tumors cells reduce tumor cells survival [103]. Uncontrolled activation of ER stress leads to growth arrest and apoptosis of cancer cells [104, 105]. Further, it was shown that ER stress can contribute to overcoming chemoresistance by increasing autophagy and apoptosis in cancer cells [106, 107]. Provoking severe ER stress can activate cancer cells suicide mechanisms through autophagy and apoptosis and has been proposed as an effective mechanism for cancer chemotherapy [108, 109]. Interestingly, Sestrin2 attenuates ER stress and ER stress-mediated apoptosis but expedites autophagy [51]. As mentioned, ER stress can increase inflammation and stimulate oncogenic pathways in the first stages of tumorigenesis. In the later stages, severe ER stress can decrease tumor cells survival, however, mild to moderate ER stress can maintain tumor cells viability by increasing autophagy (Fig. 2).

## Sestrin2 modulates the immune response

Tumor cells develop several mechanisms to evade immune surveillance [110]. Augmentation of immune response particularly T cells-relate response showed promising results in cancer treatment [110]. Herein, an immune-checkpoint blockade that can remove functional constraints and maintain effector T cells response brought satisfactory efficacy in cancer immunotherapy [111].

It was demonstrated that Sestrin2 can profoundly mitigate inflammation by inhibiting macrophage response [66]. Recently, it has been illuminated that Sestrin2 can also affect other immune cells. Interestingly, Sestrin2 modifies immune response and can induce a senescent-like phenotype in CD8+ T cells by downregulating their T cell receptor (TCR)-mediated immune response and increasing natural killer (NK) cells-like response in CD8+ cells [112]. Furthermore, Lanna et al. showed that Sestrin-mediated activation of mitogen-activated protein kinase (MAPK) suppresses T cells response similar to what happens in T cells in old humans or mice [113]. It was shown that Sestrins are major suppressors of T cells response and their deletion enhances T cells response [113]. Furthermore, it was uncovered that Sestrin2 can inhibit the cytotoxic and tumoricidal activity of NK cells by activating the AMPK/mTORC1 signaling pathway. Inhibition of mTORC1 by Sestrin2 weakened NK cells function against ovarian cancer either in vivo or in vitro [114]. The immune-suppressive property of Sestrin2 can contribute to immune evasion characteristics of cancer cells and should be considered for cancer immunotherapy.

## Protective effect of Sestrin2 against cancer

Most of the previous studies, either clinical or preclinical, revealed that Sestrin2 can protect against different cancers and several molecular mechanisms were shown to be involved in such effect.

Chen et al. reported that Sestrin2 has a lower expression in the tissue samples of patients with non-small cell lung cancer, compared with non-cancerous lung tissues [115]. Furthermore, lower expression of Sestrin2 was correlated with poor differentiation of tumor cells, advanced TNM stage, lymph node metastasis, and shorter overall survival [115]. Wei et al. observed that both human colorectal cancer tissues and cell lines have lower expression of Sestrin2, compared with normal colorectal tissue, polyps, and adenomas [18]. Also, lower expression of Sestrin2 was correlated with advanced tumor stage, lymphatic invasion, lymph node metastasis, vascular invasion, and liver metastasis and predicted shorter overall survival and disease-free survival [18]. Similarly, it was reported that Sestrin2 has a lower expression in hepatocellular carcinoma tissue, compared to non-cancerous liver tissue. Meanwhile, Sestrin2 expression decreases in higher stages of the tumor and positively correlates with patients’ survival [116]. Furthermore, lentiviral-mediated upregulation of Sestrin2 has been associated with inhibition of pancreatic cancer cells proliferation, invasion, and migration in PANC-1 and CFPAC-1 cell lines [117].

It was shown that Sestrin2 expression increases in DSS-induced colitis in mice, inhibits mTORC1 and ER stress, and finally facilitates the resolution of inflammation and the healing process [65]. Meanwhile, it was shown that Sestrin2 is controlled by P53 to inhibit mTORC1 and prevent colitis-associated colon cancer [65]. The study indicated that Sestrin2 is downregulated in human colon cancer tissue and SENS2^−/−^ is associated with increased tumor growth and chemoresistance in colitis-associated colon cancer in mice [65]. Consistently, it was shown that activation of the AMPK/mTORC1 pathway by Sestrin2 leads to activation of caspase 3, 7, and 9 and increases apoptotic cell death in colorectal cancer [118]. Interestingly, Wang et al. reported that gastric cancer and colorectal cancer downregulate an E3 ubiquitin ligase RING finger protein 167 (RNF167) and upregulate a deubiquitinase STAMBPL1 that can prevent the ubiquitination of Sestrin2, decrease Sestrin2-GATOR2 interaction and increase mTORC1 signaling [119]. Consistently, knockout of STAMBPL1 and subsequent increase in Sestrin2 ubiquitination markedly inhibited xenograft tumor growth [119].

Importantly, Sestrin2 markedly decreased HIF-1α accumulation in colorectal cancer, even in response to a hypoxic condition or in response to cobalt chloride (CoCl_2_) as a hypoxia-mimetic agent [57]. Sestrin2 depends on AMPK to enhance the activity of propyl hydroxylase. Propyl hydroxylase converts HIF-1α to OH- HIF-1α and accelerates its degradation [57]. HIF-1α downregulation by Sestrin2 decreased VEGF which is crucial for intra-tumor angiogenesis [57]. HIF-1α downregulation also decreased the expression of glucose transporter 1 (GLUT1) and lactate dehydrogenase A (LAHA) which can impair aerobic glycolysis of tumor cells [57]. Further, Yan et al. indicated that rosemary extract can increase apoptosis and decrease tumor size in the mice model of colon cancer by enhancing the expression of Nrf2 and Sestrin2 [120]. Likewise, Wei et al. revealed that Sestrin2 is downregulated in colorectal cancer and viral vector-mediated upregulation of Sestrin2 reduces colorectal cancer cells proliferation, migration, and colony formation in HCT116 and SW620 cell lines [121]. Viral vector-mediated upregulation of Sestrin2 also decreased tumor size in a mouse xenoplant model [121]. Importantly, they found that Sestrin2 inhibits the Wnt/β-catenin pathway, thereby reducing cancer cells stemness through downregulation of sex-determining region Y-Box 2 (Sox2), octamer-binding transcription factor 4 (Oct4), Kruppel-like factor 4 and c-myc [121]. Cancer stemness crucially potentiates cancer cells self-renewal, promotes their ability to metastasize, maintains their microenvironment, and enhances their potential to resist chemotherapeutic agents [122]. Hence, Sestrin2-mediated attenuation of cancer cell stemness can greatly advance cancer chemotherapy.

Sestrin2, via activation of P38 MAPK, increased the expression of tumor necrosis factor receptor superfamily member 6 (FAS receptor) in A375 and A875 melanoma cell lines and induced their apoptosis [123]. Sestrin2 also enhanced the expression of tumor necrosis factor receptor 1 (TNFR1), related FAS, and tumor necrosis factor-related apoptosis-inducing ligand (TRAIL) receptors to induce apoptosis in the lung adenocarcinoma cells [124]. Sestrin2 stimulated lysosomal degradation of X-linked inhibitor of apoptosis protein (XIAP) to promote receptor-mediated apoptosis of lung adenocarcinoma cells [124]. Similarly, it was shown that Sestrin2 activates apoptosis and mediates the anti-tumor effects of fisetin on human head and neck cancer cells. It suppressed the mTORC1/myeloid cell leukemia 1(Mcl-1) pathway to promote apoptosis in head and neck cancer cells [125]. Mcl-1 is a Bcl-2 family protein and exerts anti-apoptotic effects, hence, Sestrin2 can facilitate apoptosis by downregulating it [126]. Furthermore, SESN2 knockdown strongly reversed fisetin-induced apoptosis in cancer cells [125]. Hence, Sestrin2 is a strong regulator of apoptosis in cancer cells and augments death signals, and downregulates negative regulators of apoptosis in cancer cells.

It was shown that prostate cancer cell lines such as PC3, LNCaP clone FGC, and DU145 have lower expression of Sestrin2, compared with normal prostate epithelial cells [127]. Further, higher expression of Sestrin2 decreased cancer cells proliferation and enhanced their sensitivity to ionizing radiation [127]. Likewise, it was observed that Sestrin2 has a lower expression in bladder cancer, compared with normal neighboring tissue [93] and increased expression of Sestrin2 can inhibit human bladder cancer cells growth [92, 93].

Likewise, overexpression of Sestrin2 was associated with the downregulation of mTORC1 and HIF-1α and increased autophagic flux and apoptosis in osteosarcoma cancer cells [94]. Activation of the Sestrin2/LKB1/AMPK axis attenuated the deleterious effects of mTORC1 signaling and increased nasopharyngeal cancer cells death [89]. Likewise, overexpression of Sestrin2, in an AMPK-dependent manner, improved radiosensitization of breast cancer and colon cancer cells and these effects were abrogated after SESN2 silencing [128, 129]. Shin et al. reported that both Sestrin2 and mTORC1 have higher expression in human endometrial cancer, compared with adjacent normal tissue. Meanwhile, higher expression of Sestrin2 predicted significantly shorter overall and disease-free survival of patients in this study [130]. Interestingly, it was shown that Setrin2 overexpression is significantly correlated with the overactivation of the mTORC1/P70S6K/S6 pathway in human endometrial cancer [130]. Indeed, Sestrin2 overexpression is a compensatory mechanism that attempts to inhibit the mTORC1/P70S6K/S6 pathway in cancer cells and prevent the positive effects of mTORC1 on cancer cells proliferation. Furthermore, Sestrin2, in a mTORC1-dependent manner, inhibited the production of reactive oxygen species (ROS) in cancer cells [130]. Similar to mTORC1 inhibition by rapamycin, lentiviral overexpression of Sestrin2 decreased tumor cells proliferation, EMT, and migration [130]. Also, knockdown of SENS2 inversely promoted the mTORC1 pathway and led to tumor cells proliferation and migration in this study [130]. These findings reveal that Sestrin2 upregulation can be a compensatory response to attenuate the detrimental effect of several signaling pathways and contributes to overcoming resistance to radiotherapy or chemotherapy.

Previously, it was mentioned that Sestrin2 can attenuate inflammasome activation. Attenuation of inflammasome response by activating the Sestrin2/LKB1/AMPK axis inhibited the growth of breast cancer cells [131]. Inflammation is a key activator for numerous oncogenic pathways [132]. Activation of inflammasome/IL1β promotes breast cancer angiogenesis and progression [133]. Likewise, It was shown that inflammasome and IL1β are involved in tumor growth and metastasis and IL1 receptor antagonist significantly inhibited tumor growth and metastasis in the mice model of lung cancer [134].

Here, it is understood that Sestrin2 is involved in the pathogenesis of several types of human cancer and Sestrin2 expression can be used to determine the prognosis of cancers. Furthermore, increased expression of Sestrin2 generally helps cancer chemotherapy and overcomes resistant tumors.

## Sestrin2 promotes cancer cells survival under specific conditions

However, most of the published studies claimed that Sestrin2 decreases cancer cells survival and protects against cancers, there are also studies claiming that Sestrin2 may increase cancer cells viability under specific conditions. Herein, Chae et al. reported that Sestrin2 expression is negatively correlated with the survival of patients with lung cancer [19]. The study showed that SESN2 knockdown in a non-small cell lung cancer can decrease cancer cells stemness, proliferation, and migration and improve their chemoresistance [19]. The study indicated that SESN2 silencing decreases the expression of zinc finger E-box binding homeobox 1 (ZEB1) and Snail which are needed for EMT [19]. Further, it was uncovered that ROS induced Sestrin2 expression in cancer cells and Sestrin2 also augmented Nrf2/HO-1 axis to attenuate oxidative stress. SESN2 silencing was associated with a nearly threefold increase in ROS and shortened cancer cells survival [19]. Although, it was previously mentioned that potentiation of autophagy by Sestrin2 can improve the treatment of osteosarcoma, Tang et al. showed that upregulation of Sestrin2 in extreme conditions such as severe ER stress induced by chemotherapy can act as a rescue mechanism for osteosarcoma [135]. Indeed, Sestrin2 enhances autophagy, removes the deleterious effects of ER stress, prevents cancer cells apoptosis, and promotes chemoresistance in the extreme conditions [135]. Likewise, it has been observed that Sestrin2 upregulation can be used as a defense mechanism by head and neck squamous cell carcinoma cells to protect themselves against ROS produced by radiotherapy [136]. Interestingly, radiotherapy increased miR-182-5p to downregulate Sestrin2 and augment the effect of oxidative stress on the cancer cells [136]. Furthermore, Sestrin2 knockdown significantly enhanced radiation-induced cancer cells death [136]. These findings show that downregulation of Sestrin2 can increase cancer cells vulnerability to damage and death.

Glutamine plays a pivotal role in the energy metabolism, survival, and growth of tumor cells, and higher levels of solute carrier family 1 member 5 (SLC1A5), a glutamine transporter, is expressed in tumor cells [20, 137, 138]. Increased expression of Sestrin2 during glutamine depletion protected against NADPH and ATP depletion and ROS formation in lung cancer cells by inhibiting mTORC1 [20]. It was uncovered that glutamine depletion in non-small cell lung cancer is associated with decreased synthesis of glutathione (GSH) and increased ROS production. Subsequently, ROS activated P38 MAPK and thereby activated CCAAT/enhancer-binding protein β (CEBPβ), a transcription factor, to induce the gene expression of Sestrin2 [20]. Interestingly, it was shown that Sestrin2 and mTORC2 can enhance the expression of each other in glutamine-depleted non-small cell lung cancer to improve cancer cells survival. Consistently, it was shown that combined depletion of glutamine and either Sestrin2 or mTORC2 makes lung cancer cells susceptible to energy and redox imbalance and accelerates their death in vivo and in vitro [20]. Although, it was shown that tumor cells express a lower amount of Sestrin2 before glutamine deletion, compared to normal lung epithelial cells [20]. Increased expression of Sestrin2 during glutamine depletion led to the inhibition of mTORC1 and subsequently protected against NADPH and ATP depletion and ROS formation in lung cancer cells [20]. Sestrin2 inhibited mTORC1-mediated activation of sterol regulatory element-binding transcription factor 1 (SREBP1)/fatty acid synthase (FAS) pathway to attenuated lipogenesis and also enhanced fatty acid oxidation in energy-depleted cancer cells [20]. By, decreasing mTORC1-mediated lipogenesis and protein synthesis Sestrin2 decreased ATP consumption in stress conditions [20].

It was observed that Sestrin2 expression increases during glucose limitation and enhances colon cancer cells resistance to glucose limitation [139]. Furthermore, Sestrin2 knockdown decreased human liver cancer cell survival under glucose limitation [137]. It was shown that Sestrin2 upregulated glutamine transporters SLC1A5 and SLC7A5 to increase glutamine uptake during glucose limitation. In addition, Sestrin2 enhanced glutamine-mediated activation of peroxisome proliferator-activated receptor γ coactivator-1α (PCG-1α) [137]. Sestrin2 promoted nuclear translocation of forkhead box protein O1 (FOXO1) transcription factor to upregulate PCG-1α, thereby improving mitochondrial biogenesis [137]. Hence, Sestrin2 can improve mitochondrial biogenesis and allow cancer cells to use glutamine as the source of energy during glucose limitation [137].

However, Sestrin2 can help cancer treatment in the normal condition, it may increase cancer cells chemoresistance in specific circumstances such as energy depletion, nutrient depletion, and severe oxidative stress. This effect can help cancer cells to develop mechanisms to resist chemotherapy, and radiotherapy and maintain their viability during harsh conditions.

## Conclusion and future direction

**Fig. 3:**
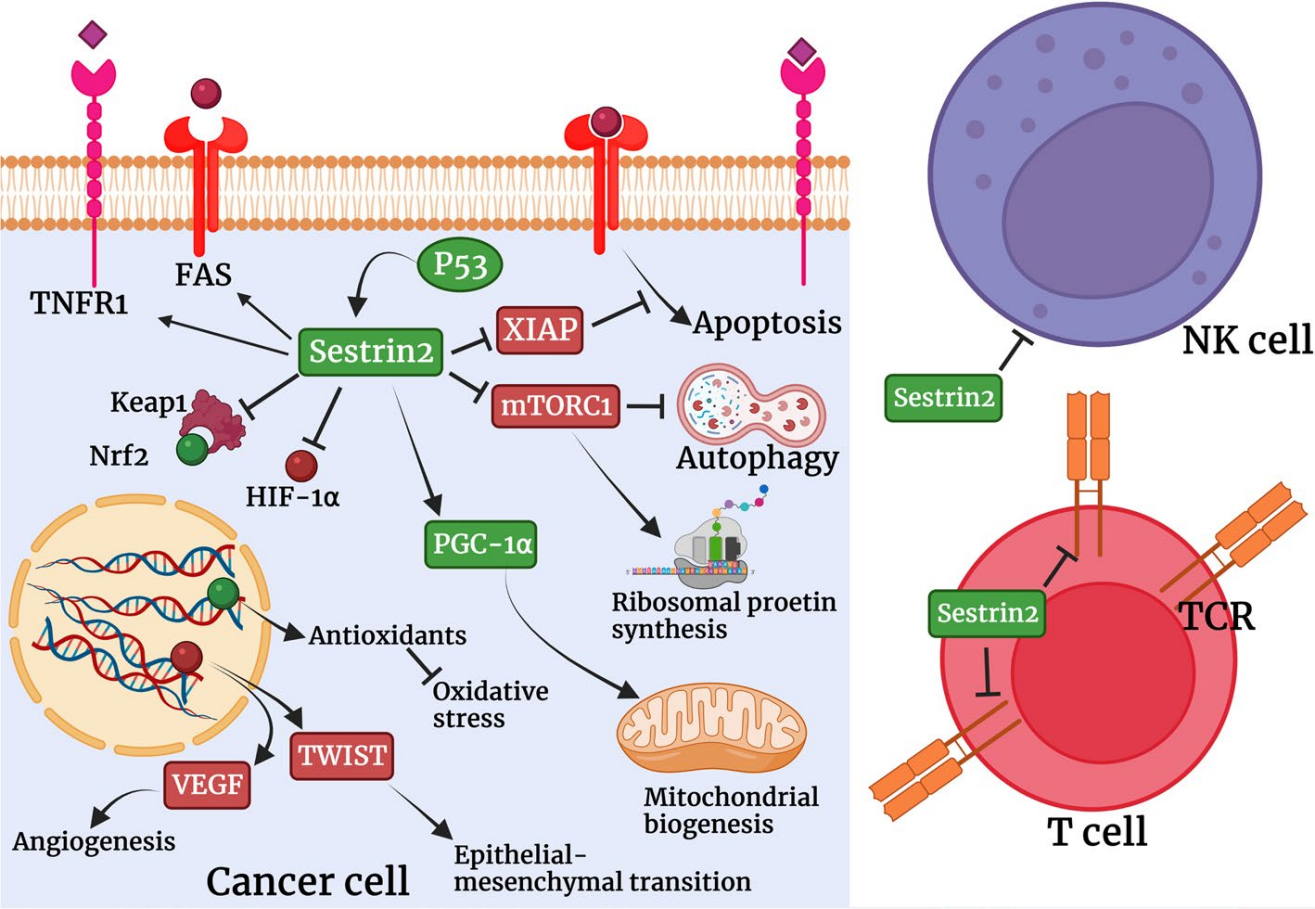
The involvement of Sestrin2 in cancer biology. Sestrin2 is vastly involved in different dimensions of cancer biology. Sestrin2 decreases immune responses particularly those related to T cells and NK cells, however, it upregulates FAS and TNFR1 to induce apoptosis. Sestrin2 also enhances antioxidant response, autophagy and mitochondrial biogenesis, and inhibits ribosomal protein synthesis, angiogenesis and EMT

Recent studies uncovered that Sestrin2 is involved in the development and progression of cancer and modulates several signaling pathways and cellular events in cancer cells. Sestrin2 can enormously affect different types of cancer, however, results were inconsistent and controversial regarding the effect of Sestrin2 on some types of cancer. Sestrin2 can be used as a prognostic factor for several types of cancer such as colorectal cancer, lung cancer, and hepatocellular carcinoma. Regarding the involvement of Sestrin2 in cancer cells apoptosis, immune evasion, invasion, metastasis, and chemoresistance, modulation of Sestrin2 expression may improve cancer chemotherapy (Fig. 3). In addition, because of its wide range of functions in cancer cells homeostasis and its involvement in the regulation of autophagy, ER stress, inflammation, and oxidative stress, it may differently affect the anti-cancer efficacy of chemotherapeutic agents. For instance, Sestrin2 improves the anti-cancer effect of bortezomib and 5-fluorouracil [140, 141]. Recently, it was shown that non-coding RNAs are heavily involved in different stages of gene expression in cancer cells and interact with oncogenes, tumor-suppressor genes, and oncogenic signaling pathways [56, 142]. New techniques such as non-coding RNAs delivery and vector systems can contribute to regulating the gene expression of Sestrin2 in cancer [118, 143]. These methods of treatment as well as direct targeting of Sestrin2 can be used in future clinical trials. All in all, Sestrin2 can be a new target for cancer chemotherapy and future preclinical and clinical studies are needed to assess the net effect of Sestrin2 in different stages of cancer development and progression.

## Data Availability

Not applicable for a review article.
